# Differing Time of Onset of Concurrent TMS-fMRI during Associative Memory Encoding: A Measure of Dynamic Connectivity

**DOI:** 10.3389/fnhum.2017.00404

**Published:** 2017-08-14

**Authors:** Colin Hawco, Jorge L. Armony, Zafiris J. Daskalakis, Marcelo T. Berlim, M. Mallar Chakravarty, G. Bruce Pike, Martin Lepage

**Affiliations:** ^1^Douglas Mental Health University Institute, McGill University, Montreal QC, Canada; ^2^Campbell Family Mental Health Institute, Centre for Addiction and Mental Health, Toronto ON, Canada; ^3^Departments of Psychiatry and Biological and Biomedical Engineering, McGill University, Montreal QC, Canada; ^4^Department of Radiology, University of Calgary, Calgary AB, Canada

**Keywords:** memory encoding, TMS-fMRI, temporal, connectivity, cognition, dorsolateral prefrontal cortex, transcranial magnetic stimulation

## Abstract

There has been a distinct shift in neuroimaging from localization of function into a more network based approach focused on connectivity. While fMRI has proven very fruitful for this, the hemodynamic signal is inherently slow which limits the temporal resolution of fMRI-only connectivity measures. The brain, however, works on a time scale of milliseconds. This study utilized concurrent transcranial magnetic stimulation (TMS)-fMRI in a novel way to obtain measures of dynamic connectivity by measuring changes in fMRI signal amplitude in regions distal to the site of stimulation following differing TMS onset times. Seventeen healthy subjects completed an associative memory encoding task known to involve the DLPFC, viewing pairs of objects which could be semantically related or unrelated. Three pulses of 10 Hz repetitive TMS were applied over the left DLPFC starting either at 200, 600, or 1000 ms after stimulus onset. Associations for related pairs were better remembered than unrelated pairs in a post-scan cued recall test. Differences in neural activity were assessed across different TMS onsets, separately for related and unrelated pairs. Time specific TMS effects were observed in several regions, including those associated with higher-level processing (lateral frontal, anterior cingulate), visual areas (occipital), and regions involved in semantic processing (e.g., left mid-temporal and medial frontal). Activity in the frontal cortex was decreased at 200 ms post-stimulus for unrelated pairs, and 1000 ms post-stimulus for related pairs. This suggests differences in the timing across conditions in which the DLFPC interacts with other PFC regions, consistent with the notion that the DLPFC is facilitating extended semantic processing for related items. This study demonstrates that time-varying TMS onset inside the MRI can be used to reliably measure fast dynamic connectivity with a temporal resolution in the hundreds of milliseconds.

## Introduction

Understanding the interactions between brain regions is critical for elucidating cognitive processing (McIntosh, 2000; Bressler and Menon, 2010). Functional connectivity provides a means to do so. When the fMRI blood oxygen level dependant (BOLD) timecourse between two regions is correlated, this suggests that neurons within these regions are communicating and/or coordinating their firing and share a functional relationship. However, the hemodynamic origins of the BOLD signal results in inherently low temporal resolution, as the BOLD signal peaks approximately five seconds after the neuronal response and is therefore not directly indicative of the timing of neuronal activity (Bartels et al., 2008; Florin et al., 2015). Neural activity, on the other hand, evolves on a time scale of milliseconds. An understanding of the rapid temporal dynamics of connectivity is critical for a full understanding of different regions in the brain work together to processes information in the world around us.

Much of our understanding of temporal processing in the brain comes from event-related potential (ERP) research. ERPs have demonstrated a temporal hierarchy to cognitive processing in the brain, with more complex functions occurring later in time. For example, there are several early peaks in response to external stimuli at 30, 100, 200, and 300 ms, representing different levels of sensory processing and early attention (Key et al., 2005). More complex processing occurs later in time. ERPs during recognition memory have notable peaks (old-vs new items) with distinct functional correlates, including an early 300–500 ms post-stimulus (familiarity), a posterior peak at 400–800 ms (recollection), and a sustained late frontal component (post-retrieval monitoring; (Friedman and Johnson, 2000; Rugg and Wilding, 2000). There is also a late-posterior negativity (extending past 1000 ms) found in response to correctly classified new items, generally appearing after response and which may represent reconstructive processing or continued evaluation of retrieval outcomes (Herron, 2007; Mecklinger et al., 2016). Critically, these temporally distinct ERP peaks also have distinct scalp topographies and distinct neural generators. Intracranial electrical recordings have allowed an even more fine-grained analysis of memory-related timing effects in the brain (Johnson and Knight, 2015). For example, time-frequency analysis in the human hippocampus during memory encoding shows theta power increases after 500 ms, while there is a peak in alpha activity around 1200–1400ms post-stimuli (Fell et al., 2011). This suggesting changes over time in how the hippocampus is processing information and interacting with the rest of the brain. Taken together electrophysiological evidence shows the importance of temporal information to fully understand cognitive processing in the brain.

A potentially interesting probe for time-specific connectivity is neuromodulation using transcranial magnetic stimulation (TMS) during fMRI (Bohning et al., 1999). TMS works by administering a magnetic pulse to a spatially specific target region of the cortex. The magnetic pulse passes unattenuated through the skin and skull and modulates activity in the underlying neuronal tissue (Sandrini et al., 2011). It has been demonstrated that when a TMS pulse is administered there is a propagation of neuronal activity to regions of the cortex which are spatially distant from the target location where the TMS coil was placed. Current evidence indicates that a TMS pulse will effect distal regions mostly during periods of active communication between the TMS target site and the distal cortical site (Ruff et al., 2006, 2008; Bestmann et al., 2008; Blankenburg et al., 2008; Feredoes et al., 2011). This allows for the intriguing possibility to use concurrent TMS-fMRI to resolve connectivity in the brain. When a TMS pulse is administered, and modulates activity in a distal region, this modulation of the underlying neural activity is accompanied by a change in relative amplitude of the BOLD signal. However, as the modulation of that distal region is related to connectivity at the *specific moment of TMS stimulation*, we can utilize amplitude changes during differing timing of TMS stimulation as a surrogate marker for the temporal dynamics of connectivity with the target site.

The purpose of this study was to demonstrate the utility of varying TMS onset timing to resolve fast temporal dynamics in functional connectivity during cognitive processing. We selected an associative encoding task know to activate the DLPFC (Blumenfeld and Ranganath, 2006; Murray and Ranganath, 2007; Blumenfeld et al., 2011; Hawco et al., 2013a). Importantly, rather than a role in general memory encoding, the DLPFC has been shown to be specifically modulated during the encoding of associative information as opposed to memory for specific objects (Blumenfeld and Ranganath, 2006, 2007; Murray and Ranganath, 2007). The ventrolateral PFC (VLPFC), by contrast, shows a more general role in memory for both item specific and associative information. It has been suggested that VLPFC activity is related to maintenance of goal-relevant item information, while the DLPFC plays a role in the organization and manipulation of goal-relevant information (Owen et al., 1996; Petrides, 1996; Blumenfeld et al., 2013). Consistent with this hypothesis, the role of the DLPFC in successful associative encoding may also be mediated by the role of the DLPFC in working memory (Blumenfeld and Ranganath, 2006; Ragland et al., 2012).

The importance of the DLPFC in associative memory has been highlighted by studies in clinical populations showing memory deficits. For example, patients with schizophrenia (SCZ) have prominent memory deficits which are strong predictors of clinical and functional outcome (Lepage et al., 2014). Memory deficits in schizophrenia have been strongly associated with hypo activation of the prefrontal cortex more so than other cortical regions (Ragland et al., 2009), with hypo-activity in the DLPFC specifically related to associative encoding as opposed to memory retrieval (Lepage et al., 2015; Ragland et al., 2015a). Interestingly, SCZ patients also demonstrate a failure to spontaneously make use of effective memory encoding strategies such as semantic organization (Iddon et al., 1998; Stone et al., 1998). However, when these individuals are provided specific instructions to make use of strategies which facilitate encoding, they normalize in both behavioral performance (McClain, 1983; Ragland et al., 2003; Brebion et al., 2004) and patterns of neural activity in the PFC (Bonner-Jackson et al., 2005, 2007; Bonner-Jackson and Barch, 2011). A similar pattern is observed in patients with prefrontal lesions; these patients show a marked deficit in memory performance which can be normalized by providing specific instructions to make use of encoding strategies such as semantic organization. (Hirst and Volpe, 1988; Incisa della Rocchetta and Milner, 1993; Stuss et al., 1994; Gershberg and Shimamura, 1995). This suggests both SCZ patients and those with PFC lesions show a pattern in which they fail to spontaneously make use of cognitive control strategies which facilitate encoding, even though their memory performance improves when such strategies are provided.

Based on this evidence, we hypothesized that the DLPFC plays a role in self-initiating memory strategies such as binding items together via semantic information. Consistent with this hypothesis, we demonstrated increased activity in the left DLPFC in a memory encoding task when participants were provided semantically related items but not explicitly instructed to consider these semantic relationships, as opposed to when semantic encoding was externally queued (Hawco et al., 2013a). A study comparing SCZ to healthy controls replicated this result of greater DLPFC activity when participants needed to self-initiate encoding strategies (Guimond et al., 2017). Group differences between SCZ were mainly driven by a sub-group of SCZ with impaired strategy use, while patients who made spontaneous use of encoding strategies showed normalized memory performance and DLPFC activity. This relationship between the DLFPC and strategy use was further demonstrated in a behavioral study using TMS; the effects of stimulation disrupting left DLPFC activity was correlated with self-reported strategy use only when related items were presented in the absence of external semantic encoding instructions (Hawco et al., 2013b). Overall, the results of these studies show that DLPFC activity is modulated by this cognitive control process of self-initiating strategy use, but not when participants are externally queued to make use of such strategies. In contrast, actually carrying out such encoding strategies has been suggested to involve the VLPFC (Kirchhoff and Buckner, 2006).

The totality of evidence of the role of the DLPFC in associative memory, related to both working memory manipulation processes and the self-initiating of strategy use, suggests the role of the DLPFC in associative memory is related to cognitive control processes. The notion of the PFC as a cognitive control region is not a new idea. For example, Miller and Cohen proposed the role of the PFC was to modulate activity across different regions of the cortex depending on internal goals relevant to cognitive processing (Miller and Cohen, 2001). Under this theory, the PFC biases responses and activity in different cortical regions during cognitive processing. Of note, regions within the PFC contain a many reciprocal long-range connections to other regions of the neocortex (Pandya and Yeterian, 1990), as well as more immediate connections to other PFC sub-regions (Petrides, 2005). The DLPFC has also been specifically linked to cognitive control processes in memory during both encoding (Ragland et al., 2015b) and retrieval (Achim and Lepage, 2005).

Given the evidence for the DLPFC as a higher-level top-down cognitive control region during associative memory encoding, we hypothesize the DLPFC will be involved with modulating activity across a variety of distant cortical regions in a task specific way. However, currently available neuroimaging methods only allow for a consideration of overall connectivity across a relatively large time scale (several seconds). We propose, based in part of the relatively fast changes in electrical brain potentials noted across ERP studies, that this high-level control process should be dynamic across relatively short time periods. That is, in order to successfully modulate activity across different regions of the cortex in a task specific manner, the DLPFC will need to interact with different cortical regions within specific windows of time related to evolving task demands. As described above, there is evidence that TMS-fMRI applied to the DLPFC will modulate activity in distal cortical regions in a task specific manner, related to ongoing in the moment connectivity between the DLPFC TMS target and the distal cortical site. For example, Feredoes et al. (2011) found that left DLPFC stimulation modulated activity in either the parietal place area or fusiform face area depending on task demands; there results suggest the DLPFC was actively maintaining sustained activity within those regions in the presence of a distractor stimuli.

We hypothesized we would be able to observe distinct changes of neural activity following different temporal windows of TMS stimulation to the left DLPFC, which were selected based on a review of ERP literature. When a TMS pulse is administered at a specific time point within a trial, we expect a modulation of neural activity in regions which are interacting with the target site at the moment of stimulation (Bestmann et al., 2008; Feredoes et al., 2011). This change in neuronal activity will result in a modulation of BOLD activity. As the hemodynamic response is approximately linear (Boynton et al., 1996; Dale and Buckner, 1997) this change will summate with ongoing BOLD responses, causing a change in signal amplitude related to changes in neural activity related to the TMS pulse. As we are varying the temporal onset of the TMS pulse, any changes in neuronal activity driven by differing TMS onsets strongly suggests evidence of the temporal dynamics of connectivity between the TMS target site (i.e., the target site was interacting with the observed region at one time point but not the other).

In order to test the possibility this hypothesis that TMS-fMRI can be used to assess dynamic connectivity, we made use of an associative memory paradigm similar to our previous studies. However, as we previously noted the DLPFC was more active in a condition in which semantically related stimuli were presented in the absence of instructions to evaluate that relationship, participants were presented with related or unrelated pairs of stimuli but were not explicitly told to evaluate such relationships. By presenting TMS at different time points across two conditions (related or unrelated pairs) we can assess dynamic connectivity changes across conditions. In terms or spatial regions with connectivity to the DLPFC, we might expect to observe changes in other frontal regions, inferior parietal (Burzynska et al., 2011; Jaeger et al., 2012), or posterior regions including fusiform (Feredoes et al., 2011) or fusiform/occipital (Achim et al., 2007; Staresina et al., 2009; Hawco et al., 2013a). Given our presumption that the DLPFC is serving as a controller region to bias activity in other regions in a task-specific context, for related pairs were might also expect to see connectivity with parts of the brain involved in semantic analysis. Due to the novelty of this approach, it is difficult to make specific hypothesis with regards to the temporal relationship between connectivity with specific regions. While fMRI studies provide grounds for hypotheses of spatial patterns of connectivity, and ERP work provides temporal patterns of activity related to specific cognitive process, we are aware of no studies to date which have merged temporal and spatial information when examining connectivity profiles within the DLPFC. As such, any specific relationships between spatial patterns of activity and specific time windows would be highly speculative. However, the timing of the effects can provide potential information as to the functional role of the DLPFC in modulating activity in other brain regions. In previous work, we related to left DLPFC to initiating encoding strategies, in this case making use of semantic relatedness to facilitate encoding (Hawco et al., 2013a,b). As this is a high-level post-processing sort of cognitive function, we would expect to see relevant activity in later time windows for object pairs which were semantically related.

## Materials and Methods

### Participants

Twenty-two right handed participants (five males, average age 21.8 years, age range 19–29) were recruited for this study. Participants completed a screening questionnaire and indicated no history of neurological or psychiatric illness or contraindications for TMS or fMRI. Two participants declined to complete the study due to discomfort associated with TMS, two participants’ session were canceled due to technical problems, and one was excluded for excessive motion (>3 mm). Seventeen data sets were included in the final analysis (four males, mean age 22.6, range 19–29).

### Memory Encoding Task

**FIGURE 1:**
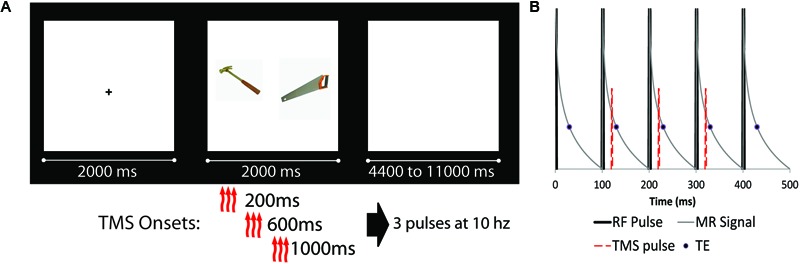
**(A)** Schematic diagram of experimental design (fixation cue, stimuli presentation, and ISI). Object pairs could be either semantically related (as the example shown) or unrelated. There were four possible transcranial magnetic stimulation (TMS) conditions: three TMS pulses at 10 Hz starting at either 200, 600, or 1000 ms after trial onset, as well as a noTMS condition. **(B)** Detailed schematic of the TMS pulse onset relative to scanner RF pulses. RF pulses are represented by thick black likes, representing slices which are acquired at 100 ms intervals. The thin gray line is a representation of the MRI signal decay, while the circle represented the data read-out time (TE = 30 ms). The TMS pulse (red dashed line) was presented approximately 20 ms after RF onset, prior to the onset of the TE, for three consecutive slices (represented as slices 2, 3, and 4 in the diagram).

The experimental task was presented using E-prime software 2.0 (build 2.0.10.182) on a PC running Windows 7. Stimuli consisted of pairs of high quality color photographs of common objects taken from the Bank of Standardized Stimuli (Brodeur et al., 2010). Half of the object pairs were semantically related to each other (e.g., axe–saw), and half were unrelated (e.g., backpack–lime). Care was taken during study design to match the quality and types of images between related and unrelated pairs. All images were of common, nameable, and manipulatable objects. Participants were instructed to examine the object pair, and judge which of the pictured objects would be larger in real life (a ‘deep’ associative encoding task). During each trial, participants saw a fixation cross for 2 s, the object pair for 2 s, and a blank screen inter-trial interval, which lasted from 4.4 to 11.2 s (**Figure 1**). Prior to the onset of the first experimental trial, two ‘TMS acclimation’ practice trials were presented, in which a pair of images depicting the same object was presented along with a train of TMS stimulation. A total of 120 experimental trials were presented during the experiment. Participants were informed that they were performing a memory study, and explicitly told that there would be a post-scan memory test for the association between objects. Thirty minutes after the end of the last encoding run, participants were given an out-of-scanner cued recall test. Participants were presented with a single object from a pair and verbally indicated which object was paired with that object during encoding, saying “pass” if they could not recall the paired object. Only the final stated match was marked, and generalizations (e.g., “it was a tool of some kind”) were not accepted as correct. The experimenter marked the participant’s response, sitting behind them to avoid any visual cues or accuracy.

### TMS Stimulation

Transcranial magnetic stimulation was administered using a Magstim Rapid2^®^ magnetic stimulator (Magstim Company Ltd., United Kingdom) with an MRI compatible focal 70-mm figure-of-eight coil (Biphasic Single Cosine Cycle with a 400 μs period). All procedures in and out of the scanner were performed in accordance with the safety specifications as outlined within the hardware documentation, and as discussed with the manufacturer. Prior to entering the MRI suite, the resting motor threshold was determined over the left primary motor cortex using the visualization method (Pridmore et al., 1998), as the lowest intensity which produced muscle activation on 5 out of 10 trials. All subsequent TMS stimulation was presented at the motor threshold. During the encoding phase inside the MRI scanner, trains of TMS consisted of three TMS pulses spaced 100ms apart (10 Hz frequency). The TMS coil was place over EEG 10/20 electrode site F3 as determined via the Beam F3 method (Beam et al., 2009), which approximately corresponds to the left DLPFC (Herwig et al., 2003a,b; Rusjan et al., 2010). The coil was placed flat against the scalp and the handle pointing 45° away from the midline toward the back of the head. A bathing cap was placed on the participants head with holes cut for the wings of the TMS coil, which helped maintain consistent contact between coil and scalp. Once the participant was on the MRI scanner bed the TMS coil was re-centered on the target site and MRI compatible padding was placed below and around the coil and participant as well as around the wire extending out of the head coil. This process provided stable positioning for the TMS coil (which was confirmed at the end of each scan by visually examining the position of the coil relative to the target site marked on the scalp) while providing maximal degrees of freedom ensuring the coil was properly perpendicular to the scalp and the handle was oriented correctly. To reduce the perceived sound of the TMS discharge, participants were provided air-conduction ear-insert headphones during the experiment, into which white noise was played at high volume which was titrated for each participant. All participants indicated the noise level of both white noise and TMS discharge was well within tolerable limits.

The onset of the TMS triplet for each trial relative to the onset of the object pair could start at 200 ms, 600 ms, or 1000 ms after stimuli onset (see **Figure 1**). For each TMS condition (including a no TMS condition), 15 related and 15 unrelated trials were presented (for a total of 120 trials). TMS pulses were administered in the gap between MRI slice excitation pulses, timed such that they never co-occurred with the MRI RF excitation pulse. Because TMS pulses had a severe adverse effect on MRI slice acquisition, the onset of the stimuli (as such, the onset of TMS) was jittered such that slices of MRI data affected by the TMS pulses were equally distributed across each condition. TMS timing was confirmed by recording the timing of TMS as a ‘response’ in E-prime, ensuring synchronization was maintained.

### fMRI Parameters

Echo-planar images were collected on a Siemens 3T Tim trio MRI using a 12 channel Head Matrix coil (TR = 3000 ms, TE = 30 ms, slice acquisition time 100 ms, 30 slices, 64 × 64 matrix, 4 mm × 4 mm × 5 mm voxels, and an interleaved slice order). These parameters were chosen such that each TMS pulse would occur during the gap between two subsequent interleaved slices. Each BOLD run was preceded by 4 volumes that were discarded to allow a magnetic steady state.

### fMRI Data Preprocessing

Prior to any further preprocessing TMS artifacts were removed from the data by interpolating data from four slices for each trial. Starting with the slice during which the first TMS pulse occurred, data was interpolated using the mean values for those slices from the previous and subsequent TR (Ruff et al., 2006; Bestmann et al., 2008; Blankenburg et al., 2008, 2010; Weiskopf et al., 2009; Feredoes et al., 2011). As slice acquisition time was set to 100ms, each TMS pulse affected a single slice. Data was then run through AFNI’s despiking procedure to resample outliers in the data. Images were motion corrected and normalized into the MNI template (with resampling to 2 mm × 2 mm × 2 mm) using SPM8^1^. White matter and CSF regressors were then manually created using masks on the MNI template of an ROI along the center of the first and second ventricles as well as a 4 mm sphere in the fourth ventricle, and six spheres (6 mm) located deep within the white matter (Supplementary Figure S1). A third order polynomial was used to remove slow drifts in the data, and mean time-course from the white matter and CSF ROIs were regressed from each voxel time-course. Data was then smoothed (10 mm FWHM) with SPM8.

### fMRI Data GLM

Preprocessed data were entered into a general linear model (GLM) in SPM8. A fisrt level GLM was performed using the canonical hemodynamic response function plus the first derivative and dispersion functions, with motion regressors included as covariates. Contrasts were created to evaluate differences in each TMS timing condition separately for each task condition, resulting in a total of six contrasts (200 ms vs. 600 ms, 200 ms vs. 1000 ms, and 600 ms vs. 1000 ms, separately for related and unrelated). By limiting the primary analysis to TMS effects across different timing conditions, the non-specific TMS effects (noise and somatosensation associated with TMS pulses) were well controlled for. Group analyses (one sample *t*-test) were performed on these contrasts, using a critical t value of *p* = 0.001 uncorrected, with an extent of 60 voxels (determined via monte-carlo simulation) resulting in a significance of *p* = 0.05 corrected for multiple comparisons. A cluster size of 100 corresponded to corrected to *p* < 0.01.

## Results

### Cued Recall Results

**FIGURE 2:**
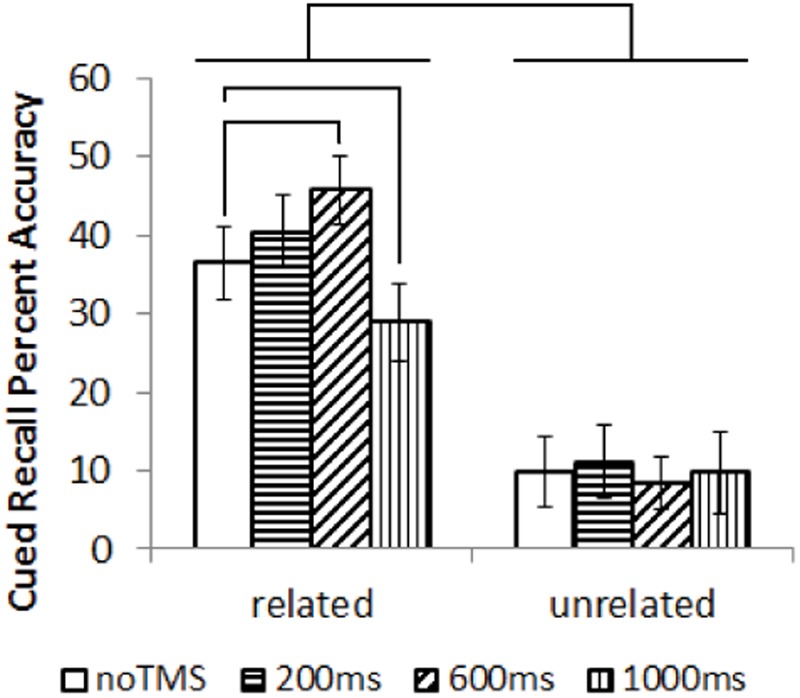
Results (mean with error bars representing standard deviation) of the cued recall test, performed outside the MRI. Lines represent significant differences between conditions (Wilcox Ranked Signs test, *p* < 0.05).

Cued recall results are shown in **Figure 2**. Data was not available for one participant. Cued recall performance for several conditions did not follow a normal distribution, showing a left skew (Kurtosis > 3). Non-parametric statistics were utilized. Overall, related trials showed significantly better subsequent cued recall than unrelated trials; Wilcox Ranked Sign test, *Z* = –3.4, *p* = 0.001. Separately for related and unrelated trials, a Friedman’s test was performed to test for an overall difference in performance across conditions. There was an overall difference in task performance for related trials (χ^2^ = 16.9, *p* = 0.001) but not for unrelated pairs (χ^2^ = 0.33, *p* = 0.95). As such, *post hoc* comparison were run on the related pairs (Wilcox Ranked Sign test). In order to limit the number of comparisons, each TMS condition was only compared to the noTMS baseline, which is the most relevant comparison. Cued recall performance for related pairs was higher in the 600ms condition compared to noTMS, *Z* = –1.963, *p* = 0.049, and worse in the 1000 ms condition compared to no TMS, *Z* = –2.103, *p* = 0.035. These results remained marginally significant (*p* < 0.1) following FDR correction.

### fMRI Results

**FIGURE 3:**
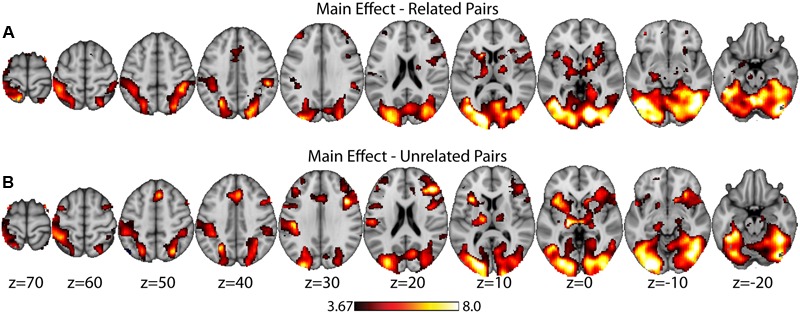
Main effects analysis (task vs. baseline) for related pairs **(A)** and unrelated pairs **(B)** in the noTMS condition. Data is presented on the MNI152 template in neurological convention (left side of the image is the left hemisphere), with MNI coordinates for each cluster shown in the bar graphs.

**FIGURE 4:**
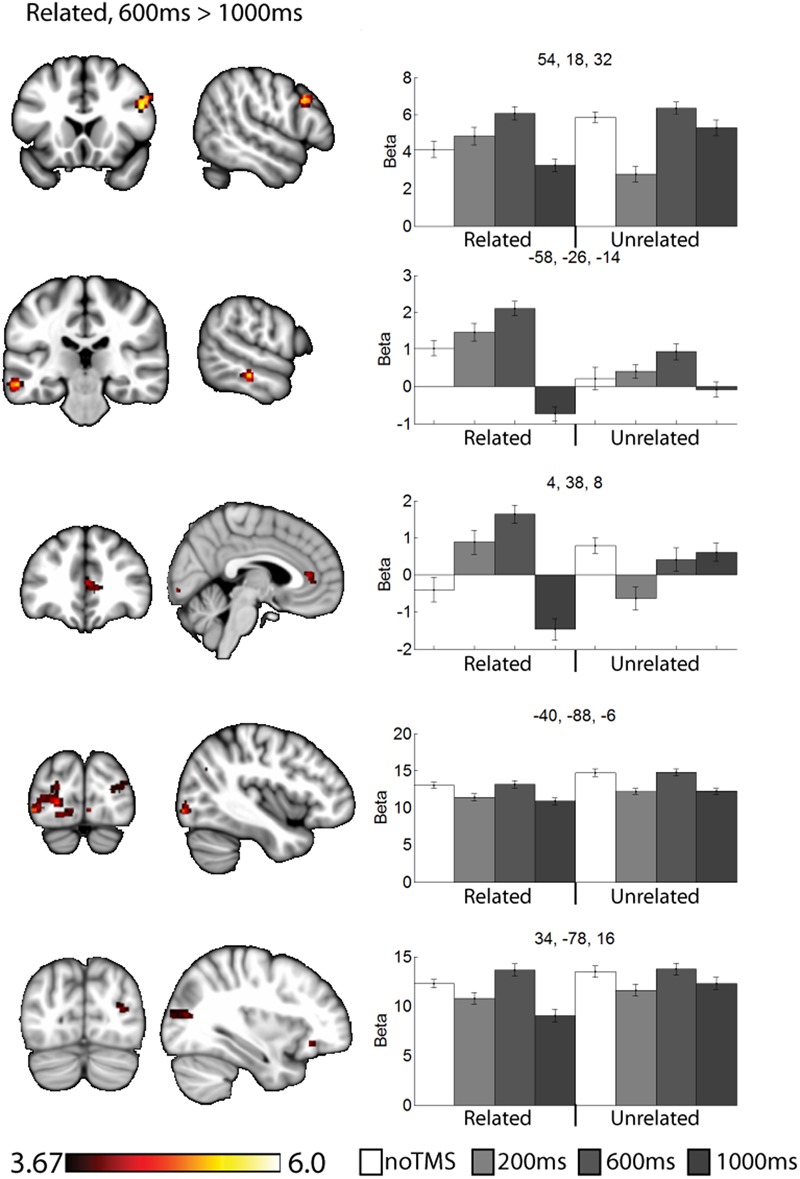
Clusters showing different neural activity at different TMS onsets (600 ms > 1000 ms) for related pairs. Bar graphs show Beta values for all TMS conditions, including no TMS and unrelated trials for comparison. Beta values were extracted for an ROI of 11 voxels centered on the peak (9 in plane and one above and one below). Error bars show standard error. Data is presented on the MNI152 template in neurological convention (left side of the image is the left hemisphere), with MNI coordinates for each cluster shown in the bar graphs.

**FIGURE 5:**
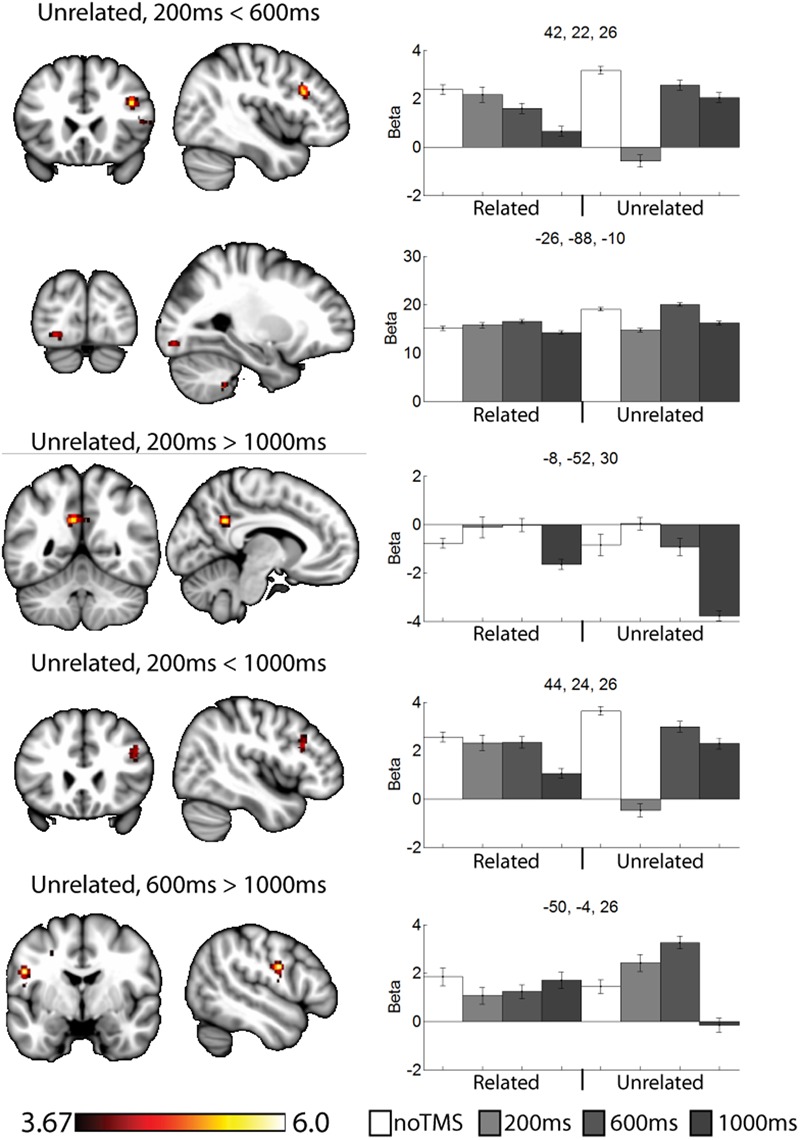
Selected clusters showing different neural activity at different TMS onsets for unrelated pairs. Bar graphs show Beta values for all TMS including no TMS and related trials for comparison. Beta values were extracted for an ROI of 11 voxels centered on the peak (9 in plane and one above and one below). Error bars show standard error. Data presented on the MNI152 template in neurological convention (left side of the image is the left hemisphere), with MNI coordinates for each cluster shown in the bar graphs.

As a data quality check, a main effects analysis was performed for the related and unrelated pairs in the noTMS condition. The results are illustrated in **Figure 3**. Both conditions showed large, widespread changed in BOLD signal relative to baseline, including visual, frontal, parietal, and sub cortical regions. Results of the fMRI contrasts between TMS timing conditions are presented in **Table 1**. Significant clusters (*t* > 3.67, extent > = 60 voxels) were observed in several contrasts, demonstrating that it is possible to see time-specific effects of TMS stimulation on activity in distal regions. Beta values were plotted for a series of selected representative clusters for related (**Figure 4**) and unrelated (**Figure 5**) contrasts.

**Table 1:** Clusters of activity in contrasts of different transcranial magnetic stimulation (TMS) timing conditions, separately for related, and unrelated trials.

Extent	MaxT	*X*	*Y*	*Z*	*BA*	Location
**Related, 200 ms > 600 ms**					
No significant clusters					
**Related, 200 ms > 1000 ms**					
No significant clusters					
**Related, 600 ms > 1000 ms**					
150	5.87	54	18	32	44	Right pars opercularis (inferior frontal)
98	5.32	–58	–26	–14	20	Left mid temporal cortex
474	4.82	–40	–88	–6	18,19	Left mid occipital and calcarine
78	4.50	4	38	8	32	Anterior cingulate
157	4.22	34	–78	16	19	Right mid occipital gyrus
**Unrelated, 200 ms > 600 ms**					
86	–5.51	42	22	26	44/45	Right middle/inferior frontal gyrus
70	–4.42	–26	–88	–10	18	Left occipital
84	–4.28	48	14	14	44	Right pars opercularis (inferior frontal)
**Unrelated, 200 ms > 1000 ms**					
144	5.61	–8	–52	30	23	Posterior cingulate
74	–4.40	44	24	26	45	Right inferior frontal/pars triangularis
**Unrelated, 600 ms > 1000 ms**					
86	5.81	–50	–4	26	4	Left motor

For related pairs (**Figure 4**), significant activity was noted in the 600 ms > 1000 ms contrast in several regions, many of which have been implicated in memory or semantic processing. The right inferior frontal cortex (pars opercularis). The left mid temporal cortex has been implicated in semantic analysis (Binder et al., 2009). The anterior cingulate is a region which has connectivity with DLPFC during associative memory related to cognitive control processes (Woodcock et al., 2015), increases activity with greater task demand (Carr et al., 2016), has been implicated in aging and memory (Martinelli et al., 2013; Gefen et al., 2015), and monosynaptically projects to the hippocampus, (Rajasethupathy et al., 2015). Occipital regions, which have been observed in similar studies (Hawco et al., 2013b; Hawco and Lepage, 2014), and the DLPFC may play a role in high-level control of visual attention (Kirchhoff et al., 2000; Katsuki and Constantinidis, 2012). Activity was also present in the right pars opercularis.

For unrelated pairs (**Figure 5**) activity was present in all three contrasts. For 200 ms > 600 ms, activity was present in the right inferior frontal and left occipital. For 200 ms vs. 1000 ms, activity was present in the posterior cingulate and left inferior frontal, and for 600 ms > 1000 ms in the motor cortex.

## Discussion

This study demonstrated that we can use time-varying TMS onsets to examine fast dynamics in connectivity during a memory encoding task. Data with methods such as ERPs, MEG, or intracranial recordings have demonstrated that there is a temporal component to cognitive processing. That is, time matters in the brain. The approach we have taken in this study is a first step toward integrating time varying TMS with the high spatial resolution of fMRI to make inferences about the temporal dynamics of connectivity in the brain. The utility of this method was demonstrated by identifying significant changes in activity to different onset times of TMS. Time-varying concurrent TMS-fMRI is a potentially powerful approach to understand evolving temporal patterns of connectivity; a necessary step to fully understand dynamic brain processes during cognition. What is more, our approach demonstrates that some connectivity patterns occur with distinct and relatively short temporal windows, which cannot be measured with traditional fMRI connectivity. While any findings should be considered preliminary at this time as this is the first study of its type and the sample size is fairly small, some interesting hypotheses can be generated based on these findings.

The results of this study can be considered to be consistent with the cognitive control model of PFC function (Miller and Cohen, 2001), as we observed specific and task-relevant interactions within distal regions at specific time windows. For related pairs, our previous work has shown a role for the DLPFC in controlling strategy use, particularly considering semantic relationships between objects when not externally prompted to do so (Hawco et al., 2013a,b). Such post-processing is highly implied by the behavioral findings in which recall was far superior for related pairs. Consistent with the notion of the DLPFC as a controller region which can bias activity within different regions, we noted clusters for related pairs in regions associated with semantic analysis such as the left mid-temporal region (Cappa et al., 1998; Noppeney and Price, 2004; Binder et al., 2009). Activity in response to related pairs was also noted in the visual cortex. For both related and unrelated trials, participants were performing the same orienting task (judging which object was larger in real life), and as such there may be no reason to expect a task-related modulation of occipital visual regions activity, though the DLPFC has been implicated in control of visual regions (Kirchhoff et al., 2000; Katsuki and Constantinidis, 2012), and we have noted similar occipital activity in a similar paradigm (Hawco et al., 2013a; Hawco and Lepage, 2014). This time varying control of occipital activity is a novel finding of this study, which we could not identify via traditional fMRI approaches. We propose that this directly demonstrates how the DLPFC can modulate activity in in high level visual areas within highly specific time windows in humans. While neurophysiological work in monkeys has suggested the DLPFC plays a role of early visual attention in these regions, our results suggest a role for DLPFC control over sustained visual attention during the task. This is consistent with the notion that the DLPFC can modulate activity in distal regions in a task relevant manner.

Several of the noted clusters of activity were in regions related to cognitive control, including the contralateral PFC, and anterior and posterior cingulate. The IFG has been implicated in memory encoding (Kirchhoff et al., 2000; Addis and McAndrews, 2006; Blumenfeld and Ranganath, 2007; Murray and Ranganath, 2007). Interesting, in the contralateral PFC activity was modulated early (200 ms) for unrelated trials and later (600 ms or 1000 ms) for related trials. The change in activity at 200 ms for unrelated and 1000ms for related both resulted in negative Beta weights (implying a decrease in activity relative to the noTMS condition). This region has been implied in memory strategy use (Kirchhoff and Buckner, 2006), and activity in the left VLPFC has been shown to be malleable with cognitive training in older adults (Kirchhoff et al., 2012). We have suggested that the left DLPFC plays a role in self-initiating memory strategy use, in conditions such as in semantically related trials from the current study (Hawco et al., 2013a,b). The timing of DLPFC modulation of VLPFC activity is consistent with this hypothesis. While the DLPFC may initiate the use of such strategies, the VLPFC plays an important role in actually carrying out such processing (Kirchhoff and Buckner, 2006). As such, we would expect VLPFC activity to be prolonged in conditions where such memory strategies are being performed. Our current results expands, upon this finding and ads a new and interesting piece of information: in cases where memory strategies are less likely to be of used, the DLPFC may actively inhibit VLPFC activity shortly after stimulus onset in order to suppress additional processing, which may be of limited benefit in such cases.

This process of semantic evaluation of related pairs is a high-level process which takes time. We suggest this is in keeping with the observed results, for unrelated pairs (were post-processing is presumably minimal, and memory performance is poor) the DLPFC mainly interacts with other regions shortly after stimulus onset. The fact that DLPFC stimulation decreased BOLD activity in these regions may suggest a down-regulation of neural activity, though interpreting the directionality of BOLD changes following a TMS probe is challenging. When the stimuli are semantically related, these relationships are evaluated as part of the encoding process. The DLPFC therefore delays its interaction and potential down-regulation with other control regions, allowing time for extended cognitive processing of the presented stimuli. We observe a somewhat similar pattern in the anterior cingulate cortex, though the changes in the 200 ms condition for unrelated were not significant. Like frontal areas, for related pairs we noted an increase in activity at 600 ms and a decrease at 1000 ms. Interesting, we noted a decrease in performance in the 1000 ms related condition, during which the DLPFC seems to be interacting with several regions. These finding suggest that the DLPFC plays a role in down-regulating activity in other cortical control regions, but the timing of such down-regulation is modulated by the demands of the task. Failure to interact or down-regulate other control regions in a timely manner has behavioral consequences. This is consistent with a cognitive control function for the DLPFC.

Some limitations of the current study should also be acknowledged. As mentioned above, the sample size was limited owing to the difficulties in collecting quality fMRI data with the TMS coil in place inside the MRI. Twenty-two participants were included in this study, resulting in only 17 useable data-sets. The majority of TMS-fMRI studies have examined less than 20 participants (Bohning et al., 1999; Nahas et al., 2001; Ruff et al., 2006, 2008; Blankenburg et al., 2008, 2010; Feredoes et al., 2011). It has been argued that a significance with a smaller sample may represent a more meaningful finding than significance in an over-powered study, which may detect effects of trivial size (Friston, 2012), however we need to be wary of over-generalizing these early findings. While the fMRI contrasts should eliminate any sensory/salience effects related to non-specific TMS effects, there is a possibility of temporal dynamics caused by the TMS as a ‘distractor’ stimulus. While it is difficult to completely rule out any such temporally specific distraction effects, it is noteworthy that in the clusters outside the occipital cortex any regions which were potentially activated by related or unrelated pairs showed distinct temporal dynamics. As such, it seems unlikely these regions were driven by pure distractor effects. The best solution to this issue would be an alternate TMS control site. However, that would require doubling the length of the experiment. That was not feasible due to the excessive number of trials required (which would be difficult for participants to complete), and hardware limitations on the number of TMS pulses which can be presented during the study. Lastly, a diode relay was not included in the fMRI setup, which may have resulted in reduced SNR from leakage current (Weiskopf et al., 2009), as was the case for many early TMS-fMRI studies. Future studies will need to incorporate a relay to improve signal inside the MRI.

## Conclusion

The findings in this study demonstrate the potential utility of time-varying TMS-fMRI to detect dynamic connectivity during cognition. We were able to show clusters in which TMS at different onsets produced differences in distal brain regions. The timing of interactions between brain regions may be of critical import for efficient and effective cognitive processing. Time varying TMS-fMRI gives us a new causally driven tool to observe with 100s of millisecond resolution of this dynamic connectivity with the high spatial resolution of fMRI, even in the context of the slow BOLD signal. Within the current study, several novel temporally specific interactions between the DLPFC and distal regions were noted. For example, the DLPFC appears to modulate activity in occipital visual regions in a time and task specific manner, which has implications for the role of the DLPFC in both early and late visual attention. Within the VLPFC, we noted an early suppression of VLPFC activity for unrelated pairs, which may suggest that the DLPFC is not only prompting additional processing related to cognitive strategies for related pairs, but actively inhibiting such processing in cases where it would presumably be of less value (e.g., unrelated pairs). This approach represents an important advancement in probing temporally specific connectivity, and is not only important for a full understanding of temporal-spatial dynamics of human brain function but may be a potential biomarker for cognitive dysfunction in psychiatric disorders and aging.

## Ethics Statement

The study was approved by the ethics committee of Douglas Hospital Research Ethics board. Participants read and signed an informed consent form prior to the study. Any questions and concerns were answered by the experimenter. Participants were made aware they could withdraw at any time.

## Author Contributions

CH was involved in the primary design of the experiment, collecting data, analysis, and manuscript preparation. ML and JA were supervising authors. ML, JA, and MB provided input on initial study design. GP provided guidance on the use of the TMS in the MRI and the MRI sequences. MC provided contributions to the analysis. ZD provided additional supervision and input into interpretation. All authors reviewed the manuscript, provided edits, and contributed to the final version of the paper.

## Conflict of Interest Statement

The authors declare that the research was conducted in the absence of any commercial or financial relationships that could be construed as a potential conflict of interest.

## Footnotes

1 http://www.fil.ion.ucl.ac.uk/spm/
