# Supplementary material for: Differing Time of Onset of Concurrent TMS-fMRI during Associative Memory Encoding: A Measure of Dynamic Connectivity
Source: Front Hum Neurosci. 2017 Aug 14;11:404. doi: 10.3389/fnhum.2017.00404 (PMC5557775; doi:10.3389/fnhum.2017.00404)
Supplement: Supplementary file 1 [file Image_1.pdf]

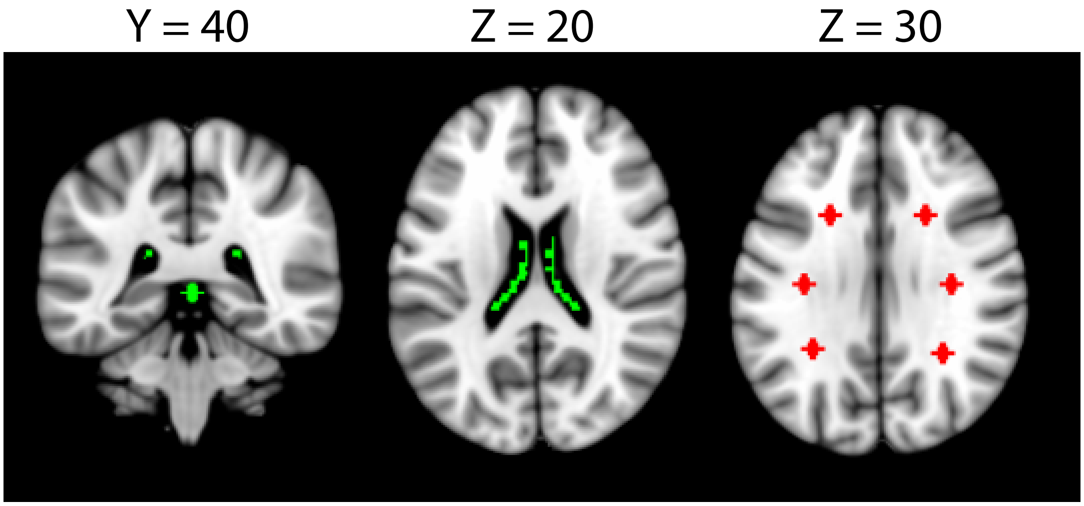

Supplementary Figure 1: ROIs used for white matter regressors (red) and CSF regressors (red) on the MNI template. Regressors were chosen such that they were deep within the white matter and CSF and therefore unlikely to overlap with gray matter signal in any participants.
